# Factors influencing change of preoperative treatment intent in a gastrointestinal cancer practice

**DOI:** 10.1186/1477-7819-5-32

**Published:** 2007-03-13

**Authors:** Roderich E Schwarz

**Affiliations:** 1 Department of Surgery, University of Medicine and Dentistry of New Jersey, Robert Wood Johnson Medical School, Division of Surgical Oncology, The Cancer Institute of New Jersey, New Brunswick, NJ 08903, USA

## Abstract

**Background:**

Postoperative assessment of indications for cancer directed surgical procedures frequently differs from preoperative plans.

**Methods:**

Specifically defined preoperative indications and postoperative results were followed prospectively over 48 months in a single surgeon academic practice, and relationships to postoperative outcomes evaluated.

**Results:**

Operations were performed on 406 patients with a median age of 61 (range: 18–90). Major operations (n = 303, 75%) involved 270 abdominal resections including pancreatectomies (37%), liver resections (23%), gastrectomies (19%), and others (21%). Preoperative curative (70%), diagnostic (38%), palliative (12%), access (9%), and non-cancer related therapy (21%) goals were in part combined in 176 patients (43%). Postoperative assessment differed from preoperative goals in 118 patients (29%). Predominant reasons were proof of benign disease (n = 35), incomplete resection (R1 or R2, n = 23), unresectability by laparoscopy (n = 21) or laparotomy (n = 21), or others (n = 18). Potential preoperative cure or palliation goals were not achieved in 37% or 15% of cases, respectively. Circumstances of changed treatment intent were specific for disease site.

**Conclusion:**

Preoperative therapeutic intent frequently differs from postoperative assessments in gastrointestinal cancer, based on shortcomings in diagnosis or therapy. Formulations of precise operative indications are recommended to optimize individual outcomes and avoid unnecessary or ineffective procedures.

## Background

Solid tumors provide most of the indications for operative therapy within surgical oncology. The majority of solid tumors reflect a malignant process, most of which in turn require complete local control through resection in order to achieve a successful long-term treatment result. Although multimodality therapy has improved treatment options and outcomes for many cancers, successful cures are rarely achieved through nonoperative modalities alone. Chemoradiation approaches to anal cancer [1] or systemic therapy for testicular cancer represent some exceptions to this rule [2]. As a result of this historically developed factum, indications to perform operative therapy are frequently assumed just due to the mere presence of apparently localized, "resectable" disease. Definitions of patient "operability" (e.g. additional comorbidity or conditions jeopardizing successful recovery from an operation) and tumor "resectability" (complete control of macroscopically identifiable disease through resection) vary widely, dependent of the quality of preoperative imaging and the individual surgeon's judgment. This is likely reflected in the fact that for many complex cancer operations, surgeon experience and volume have been found to have prognostic impact [3-7]. Many surgeons tend to resort to personal experience and subjective criteria in the process of deciding in favor of or against an operation. Goals for such therapy tend to not infrequently remain unstated, be imprecise, or become bundled such as in cases of complex tumor presentations in patients with associated symptoms, for instance due to gastrointestinal cancer. If a resection necessary to allow for a curative result cannot be performed, lesser extent procedures such as bypass operations are still commonly performed in order to prevent or ameliorate problems related to the persisting tumor [8], even if nonoperative palliative measures exist. Additionally, goals to completely eradicate the tumor mass and to treat related symptoms at the same time frequently have to be combined. However, postoperative outcomes frequently differ from stated operative goals, as true cures are sparse [9-11], and as for palliative procedures survival may be short [12], with symptom control frequently being poor [13,14] or short lived [15]. Many clinical series underreport problems related to changes in operative extent or intent, and focus instead on patient cohorts treated uniformly by resection or bypass procedures. However, one measure for treatment success of cancer operations should be the frequency of reaching preoperatively defined therapeutic goals, which is directly dependent on the precision with which these goals are formulated. The current study reflects an attempt to define specific therapeutic goals preoperatively, measure the ability of achieving these goals, and study some mechanisms that determine success or failure in this context.

## Patients and methods

The analysis is based on preoperative decisions and postoperative findings of all patients undergoing an operative procedure by a single surgeon in an academic, tertiary care cancer center setting with a clinical focus on gastrointestinal cancer. Not all patients had a specified cancer-related operative indication. Specifically defined preoperative indications and postoperative results were followed prospectively over 48 months from January 2002 until December 2005. Surgical treatment intent was classified based on five predefined categories of curative, palliative, diagnostic, access, and nonmalignant therapeutic goals. Operations were defined as of "curative" intent when designed to completely remove proven or suspected malignant disease for lasting freedom of cancer or a significant survival benefit. "Palliative" procedures had the intent to control specific symptoms caused directly by a malignant process that itself was not amenable to cure, such as malignant bowel obstruction due to peritoneal carcinomatosis. "Diagnostic" operations were to clarify an uncertain or unproven diagnosis through removal of tissue, or an unclear therapy-relevant disease extent through intraoperative imaging methods. "Access" procedures were to provide operative vascular, enteric, airway, or body cavity access for foreign devices helpful in a patient's management, such as intravenous or intraperitoneal implantable chemotherapy port placement. An operation was deemed "therapeutic" when indicated to treat a patient's nonneoplastic condition irrespective of the presence of a cancer diagnosis, such as incisional hernia repair or cholecystectomy for cholecystitis. Based on these definitions, multiple intent group assignments were specifically possible; for instance, the dual assignment "curative/palliative" was given, when preoperative clinical staging did not reveal extraregional metastatic disease, but cancer-related symptoms would justify an operation even when metastatic disease was identified intraoperatively. For elective, curative intent procedures, all patients had undergone state-of-the-art crossectional imaging by either computed tomography (favored for gastrointestinal and pancreatic malignancies) or magnetic resonance imaging (favored for hepatobiliary and soft tissue tumors); preoperative biopsies or endoscopic ultrasound examinations were utilized selectively to clarify questions important in the decision making process for operative therapy. For this "state-of-the-art" imaging, scans obtained prior to the patient's initial presentation were acceptable, as long as the defined objectives were felt to be sufficiently accomplished. These included absence of extraregional metastatic disease, and sufficient evidence for loco-regional resectability, primarily in relation to major vascular structures. Loco-regional criteria were thus organ- or site-specific. For instance, for pancreatic lesions, multiphase fine-cut helical scans were expected to delineate tumor extent, and prove freedom of superior mesenteric, vascular and portal vasculature; for hepatobiliary lesions, multiphase, fine-cut scans were expected to show intrahepatic disease extent, as well as lack of involvement of vascular structures within the hepatoduodenal ligament or at the hepatic veins. If these criteria were not answerable based on outside scans, specific scans were ordered based on disease-site protocol criteria. Clinical diagnosis and stage, as well as preoperative treatment intent were charted preoperatively. Diagnostic laparoscopy was routinely performed at the beginning of operations with curative intent for upper gastrointestinal, pancreatic, periampullary, and biliary cancers; when performed in this routine fashion, this component was not charted as diagnostic procedure. Operative findings and the procedure performed were documented postoperatively, in addition to pathology findings and postoperative outcomes. All operative cases were classified as major or minor. Major operations included any extensive visceral or radical soft tissue resection, extended lymphadenectomy, or any procedure requiring anastomotic reconstruction; in addition, emergency procedures such as cholecystectomy for gangrenous cholecystitis in a neutropenic patient were classified similarly. Minor cases included resections of smaller scope (e.g. elective cholecystectomy), nonresective/nonanastomotic procedures, extrinsic diversions, access procedures, or biopsies. Postoperative mortality was based on lethal events occurring within 30 days after operation or during the postoperative in-hospital stay if longer than 30 days. Postoperative morbidity included any untoward events deviating from normal recovery and requiring diagnostic or therapeutic intervention; events requiring intensive care, reoperations, or interventional radiological management were considered major complications. Postoperative treatment intent reassessment was carried out for each preoperative treatment goal, and successful as well as unsuccessful intent realization based on intraoperative findings or pathology results was documented. Relationships between preoperative and postoperative treatment goals, and group comparison of nominal variables were evaluated via chi square contingency analysis or Fisher's exact test, as appropriate. Variables predicting the failure to achieve a preoperative treatment intent category were examined through logistic regression analyses, employing a stepwise backward methodology. Covariates entered into this model were all demographic and clinicopathologic variables, organ site information, and all preoperative intent categories (such as curative yes/no, etc.). For the group comparison of postoperative length of stay, a nonparametric product-limit method with Peto-Peto-Wilcoxon test was chosen as described earlier [16]. Postoperative deaths were excluded from length of stay analysis. Group differences were assumed significant at p < 0.05. All analyses were performed with StatView 5.0.1 software for Macintosh computers (SAS Institute Inc., Cary, NC).

## Results

### Patient demographics and operative treatment

During the defined time interval, operations were performed on 406 patients. There were 198 males (49%) and 208 females (51%), with a median age of 61 (range: 18–90). Three hundred and two patients had a final diagnosis of cancer (74%), of whom 73% underwent a major operation. The proportion of major operations in patients with a benign diagnosis (n = 104, 26%) was 80%. Major operations were altogether performed in 303 individuals (75%) and predominantly involved transabdominal resections (n = 270). The distribution of resections among organ sites included pancreatectomies (37%), liver resections (23%), gastrectomies (19%), and others such as esophagectomies, intestinal or soft tissue resections (together 21%). The ratio of malignant versus benign diagnoses undergoing major resection did not show significant differences among various organ sites; 73% of pancreatectomies, 79% of hepatobiliary resections, 78% of gastrectomies, and 69% of other organ resections were performed for cancer. Thirty-four operations were performed as emergencies (8.4%); of all cancer-directed procedures, 6% were emergencies, compared to 15.4% of operations for a nonmalignant process (p = 0.006). The utilization of preoperative diagnostic studies and laparoscopy varied by organ site (Table 1).

**Table 1:** Utilization for standard diagnostic procedures, by intraabdominal organ site

Organ site	CT	MRI	EUS	Laparoscopy
Esophagus (n = 10)	100	0	40	60
Stomach (n = 72)	93	10	28	56
Small bowel (n = 18)	83	6	6	33
Colon (n = 17)	88	12	0	6
Rectum (n = 19)	95	21	44	11
Liver (n = 84)	83	83	2	15
Bile ducts (n = 47)	96	43	13	53
Pancreas (n = 125)	95	32	40	76
Retroperitoneum (n = 12)	100	67	8	17
Soft tissues (n = 10)	80	40	10	10

Numbers reflect % (rounded) of patients within the disease group who had undergone the diagnostic test. The patient numbers within diagnostic sites do not add up to 406, since extraabdominal and hematologic diagnoses were excluded, and 75 patients carried combined diagnoses of different organ sites.

### Preoperative treatment intent

The preoperatively defined treatment plan listed curative goals in 70%, diagnostic in 38%, palliative in 12%, access-related in 9%, and non-cancer related therapy goals for 21% of all patients. Various treatment goals were combined in 176 individuals (43%).

Procedures in patients with preoperative curative goals differed from those without curative goals, with regard to resections (82% vs. 30%), bypasses or diversions (3% vs. 9%), and minor procedures (15% vs. 61%, p < 0.0001). Similarly, nineteen percent of patients with preoperative palliation goals underwent a bypass procedure (versus 3% of those without palliation needs), and 74% a resection (versus 65% of those without palliation needs, p < 0.0001). Six percent of emergency operations still carried curative plans, while 41% were performed in order to achieve palliation. The highest frequencies of curative indications were created for pancreatic (95%), esophageal (90%), gastric (84%), small bowel (82%), retroperitoneal (80%), and hepatobiliary (74%) diagnoses. Conversely, palliative goals were most prevalent in patients with pelvic (45%), colon (44%), gynecologic (33%), gastric (29%), and small bowel disorders (27%). Seventy-six percent of access goals were carried out in minor procedures, while 3% of major cases were to have an access procedure component (p < 0.0001). Cancer was ultimately confirmed in 64% of patients with a planned diagnostic component to the operation, while 33% of cancer patients underwent an operation with a diagnostic goal (p = 0.0001). Treatment goals unrelated to the cancer were to be addressed in 8% of patients at the same time of attempting a curative operation.

### Postoperative outcomes

The overall postoperative mortality for the entire cohort was 4.7%, based on 19 lethal events after 406 operations. Mortality figures did not differ significantly when compared by diagnostic group or operative extent (Table 2). However, the death rate after emergency operations (11.4%) exceeded that after elective operations (4.0%, p = 0.04).

**Table 2:** Outcomes by diagnostic group and procedure extent

	Total cohort	Cancer	Benign	Major operation	Minor operation
Major complications (%)	13	14	11	17*	1*
Mortality (%)	4.7	5.3	2.9	5.6	1.9
LOS, d (range)	7 (0–100)	7 (0–60)	6 (0–100)	8 (1–100)*	1 (0–33)*

• p < 0.0001 (Major versus minor operation)

Postoperative complications were encountered in 98 patients (24%); fifty-three of these were classified as major, representing 13% of all patients. Major complications were associated with major operative procedures, but not related to diagnostic group (Table 2). Major complications occurred in 19% of patients after pancreatectomy, 15% after hepatectomy, 18% after gastrectomy, and 19% after other major resections (p = N.S.). Twenty four percent of emergency operations led to a major complication, compared to 12% of nonemergent cases (p = 0.058). The median postoperative length of stay was 8 days (range 1–100) after major operations, and 1 day after minor procedures (0–33; p < 0.0001). A cancer diagnosis did not significantly influence the hospital stay (7 versus 6 days, p = N.S.), but an emergency setting did, both for major (9 versus 8 days, p = 0.01) and minor (6 versus 0 days, p = 0.04) cases.

### Postoperative reassessment of treatment intent

**Figure 1:**
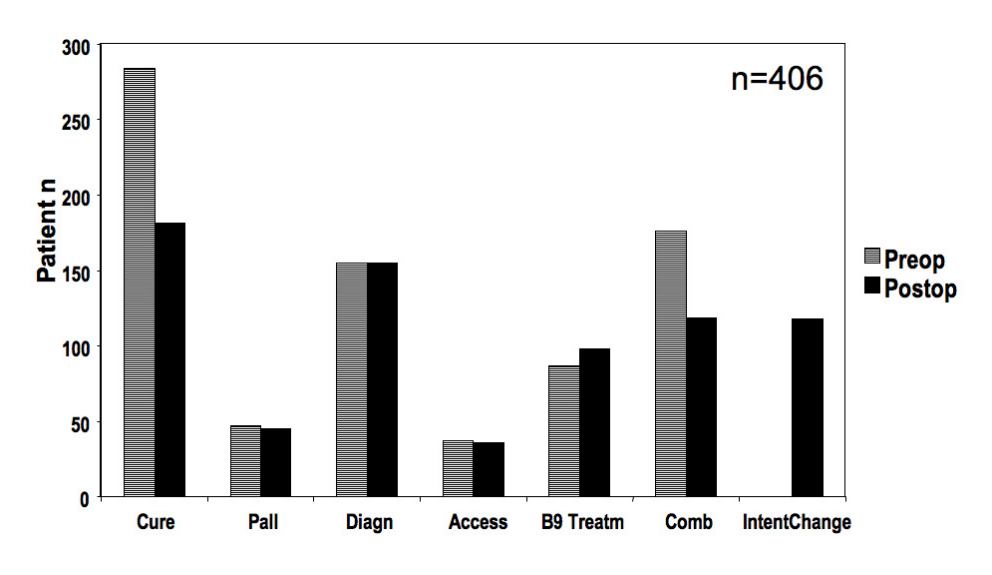
Preoperative versus postoperative assessment of operative intent. Pall = palliative. Intent. Diagn = diagnostic intent. B9 Treatm = benign disease therapeutic intent. Comb = combined intent. The last bar represents the total number of patients with an intent change in any category, by definition a postoperative event.

**Figure 2:**
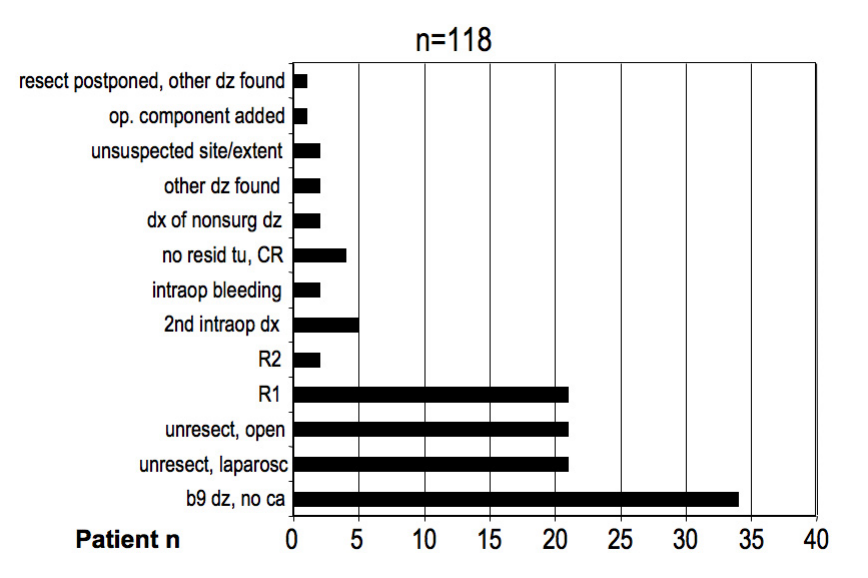
Frequency of reasons for a postoperative change of the preoperative intent. dz = disease. dx = diagnosis. CR = complete response. R2 = R2 resection. R1 = R1 resection. b9 = benign.

Postoperative assessment, based on intraoperative findings and histopathological results, differed from preoperative goals in 118 patients (29%). The majority of cases to account for this difference were those of preoperative curative intent, but not classifiable as curative postoperatively (Figure 1). Potential preoperative cure goals were not achieved in 37% of cases. Predominant reasons for any postoperative intent change were proof of benign disease after a possible cancer had been assumed preoperatively (n = 35), incomplete resection (R1 or R2, n = 23), unresectability by laparoscopy (n = 21) or laparotomy (n = 21), or others (n = 18), as listed in detail in Figure 2. Most "other" causes for curative intent changes could be traced to uncertainty of diagnosis or disease extent; in only two patients, intraoperative bleeding prevented the completion of a curative procedure. Reasons for a change of preoperative curative intent were strongly dependent on the preoperative biopsy status, as listed in Table 3. All of the 35 patients in the "benign disease proven" category (i.e. postoperative pathology findings revealing a benign process in a patient suspected to have cancer) still required an operation for diagnostic clarification and other therapeutic reasons (i.e. resection of a noninvasive intraductal papillary mucinous neoplasms of pancreas). While all 21 patients with a positive laparoscopy can be considered appropriately treated, 13 of 23 patients with R1/2 status (57%) and 16 of 21 patients found unresectable at open laparotomy could have been spared an open operation under ideal circumstances, primarily if a proper endoscopic ultrasound had been performed, if laparoscopy had been performed, if a laparoscopy would not have been false negative, or if the open operation was merely conducted for a curative intent reason without palliation needs. These 29 individuals comprise 7% of the entire cohort, or 25% of 118 patients with postoperative intent change; conversely, 75% of operations with intent change were still justifiable.

**Table 3:** Postoperative curative treatment intent change and preoperative biopsy status (in %)

	Cancer was suspected, but benign disease proven (n = 35)	R1/2 resection (n = 23)	Unresectable (n = 42)
No preoperative biopsy obtained	34	0	17
Preoperative biopsy failed to demonstrate cancer	62	28	21
Positive preoperative biopsy	3	72	62
Totals (%)	100	100	100

p < 0.0001

Of 136 patients with a potential preoperative curative intent who had been categorized preoperatively according to the likelihood of a suspected cancer diagnosis, 92 (68%) ultimately had a cancer confirmed. In these, the ability to predict cancer followed a highly correlative pattern: of 23 patients with a lower possibility for cancer (i.e. cystic lesion of pancreas with wall abnormality), only one was found to have invasive cancer (4%); 23 of 44 patients (58%) with a likely cancer (i.e. solid mass), and 68 of 69 patients (99%) with preoperative biopsy proof were ultimately confirmed to have a malignancy. The false positive event was a patient with biopsy-proven gastric cancer who was found to have complete resolution of endoscopic abnormalities prior to his planned gastrectomy.

The frequency of unresectability findings (by metastases and local extent) differed depending on the organ to be resected, limiting planned biliary and gastroesophageal resections most often as listed in Table 4. Multivariate analysis variables associated with the failure to perform a curative resection, based on the confirmation of unresectability or R1/2 resection, are listed in Table 5. They represent certain organ sites with high metastasis potential, and complex clinical presentation with combined palliation and cure intents. Some circumstances of changed treatment intent were specific for disease site; the following p values represent comparisons to other disease sites. Among pancreas cases, 40% of changes were noncancerous and 26% had R1 resections (p = 0.006); biliary changes were due to open unresectability in 51% (p = 0.002); patients with liver lesions experienced a lesser yield from laparoscopy, and a higher rate of "other" reasons for intent change, including the identification of a second intraoperative diagnosis and a complete response after preoperative chemotherapy (p = 0.01); and stomach cancers had a higher unresectability rate (42% of changes) identified by laparoscopy, and a lower rate of benign disease proof (p = 0.01); finally, soft tissue resections displayed a higher rate of "other" intent changes, due to unsuspected tumor origin or extent (p = 0.019).

**Table 4:** Frequency rank of unresectability findings by organ resection sites, based on laparoscopy and open laparotomy results

Rank	Unresectable by laparoscopy	Unresectable by open laparotomy	Unresectable (combined)
1	biliary 20% (4/20)	biliary 15% (3/20)	biliary 35% (7/20)
2	stomach 16% (8/51)	retroperitoneal 13% (1/8)	esophagus 22% (2/9)
3	esophagus 11% (1/9)	esophagus 11% (1/9)	stomach 20% (10/51)
4	small intestine 11% (1/9)	colon 10% (1/10)	pancreas 14% (15/110)
5	pancreas 5% (6/110)	rectum 9% (1/11)	retroperitoneal 13% (1/8)
6	liver 2% (1/49)	pancreas 8% (9/110)	small intestine 11% (1/9)
7		liver 6% (3/49)	colon 10% (1/10)
8		stomach 4% (2/51)	rectum 9% (1/11)
9			liver 8% (4/49)

**Table 5:** Multiple logistic regression of variables associated with the failure to perform a curative resection (confirmation of unresectability, or R1/2 resection)

**Covariate**	**p Value**	**OR**
Biliary case	< 0.0001	5.83
Combined purpose preoperatively	< 0.0001	3.60
No benign treatment purpose preoperatively	0.0002	19.67
Stomach case	0.0054	2.82
Pancreas case	0.0108	2.36

OR = odds ratio

Combined purpose preoperatively: multiple indications (such as cure, access, benign condition treatment) existed simultaneously

No benign treatment purpose preoperatively: a benign condition treatment indication (such as cholecystectomy for gallstones, incisional hernia mesh repair, etc.) did not exist aside from the curative indication

Potential preoperative palliation goals were not achieved in 15% of cases. Planned operations with palliative components (n = 47) did not succeed in seven patients for the following reasons: unresectable malignant disease (n = 4, of which 3 were later palliated by endoscopic maneuvers), no malignant disease process (n = 2, both with symptoms relieved), and persisting early postoperative symptoms despite an accomplished resection (n = 1). In five instances without specific palliation needs stated preoperatively, a procedure was considered palliative based on intraoperative findings for the following reasons: unresectable pancreatic cancer where symptoms were strongly suspected to develop (preemptive bypass, n = 2), cancer recurrences detected intraoperatively and linked to preoperative symptoms (n = 2), and unresectable esophageal cancer with gastrostomy and jejunostomy access to enable chemoradiation (n = 1). Most palliative goals were created for gastric cancer (n = 18, 38%), colon cancer (n = 8, 17%), pancreatic cancer (n = 5, 11%) and pelvic malignancies (n = 5, 11%; p < 0.0001). Postoperative palliation assignment were most common in the same diagnostic groups (p < 0.0001).

### Postoperative reassessment of stage assignment

Of 266 patients with preoperative (clinical) stage assignment, 121 (45.5%) ultimately were classified into a different pathohologic stage category postoperatively. Patients undergoing a curative intent procedure were restaged 50.4% of the time. Among these, some significant deviations were noticed based on the organ site of disease. Compared to their respective controls, liver resections led to stage reassignment in only 26.9% (p = 0.0001), while biliary (71.4%, p = 0.018) and pancreatic resections (58.7%, p = 0.04) did so more frequently. Other organ sites were linked to stage reassignments in 77.8% (esophageal), 60.4% (gastric), or 55.6% (colonic) of cases, but differences to controls were not significant.

## Discussion

The presented analysis reflects an attempt to specify operative indications for all surgical procedures performed in a tertiary cancer center setting by a surgical oncologist with a practice focus on gastrointestinal cancer. The results show that under current practice standards, preoperative therapeutic intent still frequently differs from postoperative assessments. Discrepancies appear to be disease site specific, and relate to shortcomings in diagnosis or therapy. However, situations of apparent changes of indication assessment cannot be equated with not indicated or improperly performed operations. Only 7% of all patients, or 25% of those with a postoperative intent change would have benefited from no operative procedure or from a different operation. Changes in indication assessment, primarily observed in operations with an initial curative intent, much rather reflect the imprecision of diagnostic and staging information which still accompanies today's operative decision making, despite an obvious improvement in diagnostic imaging over the past decade. The experience presented reflects findings and challenges of a gastrointestinal oncology practice, which includes some conditions that can be localized to an interface between benign and malignant diseases, such as pancreatic masses or biliary strictures. Accordingly, disease-specific reasons for intent changes are most likely to emerge for diseases most prevalent in this practice, namely pancreatic, hepatobiliary, and upper gastrointestinal cancer, and benign conditions within the same organ sites. Despite this, the overall results, irrespective of disease-specific implications, deserve further comment.

Patients presenting with a localized mass lesion, without evidence for extraregional or unresectable disease, generally are candidates for surgical resection. In contrast to nonoperative oncological therapy, a tissue diagnosis is frequently not required in order to proceed, especially if surrogate tests such as serum tumor marker levels are abnormal. Irrespective of the presence of an invasive neoplasm or a precancerous or benign process, an operation is indicated, as diagnostic clarification, cancer cure, or therapeutic solution to a noncancerous process can all hereby be achieved. Preoperative intents, therefore, may well be reasonably combined, based on acceptable shortcomings of available information that is best clarified through the operation itself and not through additional expensive tests. In cases of symptoms caused by a malignancy, preoperative intents frequently have to be combined. It does not appear sensible to divide cases prior to an operation into just either curative or palliative, as attempted by McCahill *et al*., [17], since both curative and palliative potential may coexist preoperatively in some patients, as demonstrated before for gastric cancer [18]. It is however important to stress that a noncurative procedure does not automatically equal a palliative assignment. Again, in a clinical gastric cancer series, 19% of gastrectomies were considered noncurative, but only 48% of those were apparently performed for palliative reasons; the resulting survival was significantly shorter after palliative resections than after noncurative, nonpalliative gastrectomies [19]. This supports the strategy to delineate curative, palliative, and other operative needs and apparent potential preoperatively, and balance operative options with available nonoperative approaches such as endoscopic palliative stenting. For cancers with curative potential, combined preoperative indications apparently have important implications. In this series, patients with combined preoperative goals had a lesser likelihood for a successful curative procedure, especially if no unrelated benign condition was to be addressed operatively at the same time. Although not entirely obvious, it is likely that patients are offered additional components such as cholecystectomy or incisional hernia repair when less symptomatic, either due to lesser tumor burden, or because of a better performance status. Combined intent categories may thus also represent surrogates for other relevant outcome predictors.

Unresectability (in 42 patients) and positive margin resections (in 23 individuals) have been the dominant reasons for a failure to accomplish a potentially curative procedure in cancer patients within this series. Modern imaging has significantly improved the ability to predict resectability. As example, 3D computed tomography has been reported to accurately predict general resectability of pancreatic cancers in 79%, and of other periampullary cancers in 98%; R0 resection was predicted in 73% and 86%, respectively [20]. Diagnostic laparoscopy has greatly reduced the frequency of unnecessary laparotomies in patients with metastatic cancer. Biliary cancer, in this series the leading diagnosis for failure to resect, allowed for only a 31% resectability in a series of 100 patients; of all unresectable patients, 51% were identified by laparoscopy, but 49% only through an open procedure [21] The primary challenge here would appear to be an improved way to predict resectability of local, nonmetastatic disease.

The second reason for not achieving a curative outcome was positive margin resection. For essentially all gastrointestinal adenocarcinomas R1/2 resections can be equated with an exceedingly high risk of cancer recurrence, and limited overall survival. Again, R0 rates are lowest for biliary cancers; in a recent series, it was only 53%, and only R0 resections led to prolonged survival [22]. For other diseases such as gastric cancer, the outcomes after R0 resection depend on nodal disease burden, as distant recurrence and death may take place prior to any symptomatic margin recurrence in patients with advanced stage disease [23]. Even for malignancies other than adenocarcinomas, incomplete resection and positive margins have been linked to poor survival, such as retroperitoneal liposarcomas [24]. The primary challenge of this category appears to be better margin prediction through improved preoperative imaging, as well as preoperative treatment strategies to reduce the risk for incomplete resection. In this clinical series, intraoperative frozen section was liberally utilized in situations where a wider resection of a concerning margin area was feasible and would have allowed a R0 procedure. Positive margins thus primarily reflect either radial margins of specimens after committing to a resection, for which wider dissection was not deemed safe or sensible (e.g. the retroperitoneal soft tissue and superior mesenteric artery margin during pancreatoduodenectomy), or margins accepted because of an advanced disease burden (e.g. esophageal margin after gastrectomy for N3 gastric cancer). As stated earlier, intraoperative frozen section had not been utilized in mass lesions for which a resection indication was given, irrespective of an underlying malignant or benign process. The change in "curative" intent in this category is therefore not suggested to be improved by any other intraoperative means.

What then is the impact of the presented findings on this practice in the future? Disease-specific preoperative workup has been adjusted to include endoscopic ultrasonography routinely for those lesions that have been correlated with a high unresectability rate as defined through open laparotomy, such as biliary cancers or rare hepatic malignancies with a higher risk for extraregional nodal involvement. Endoscopic ultrasound has also routinely been applied to pancreatobiliary cystic lesions, to delineate which cysts are to be resected based on defined imaging criteria, and which are to be followed over time. Diagnostic laparoscopy will be performed for those cancers with palliative indications, for which alternative palliation means are to be preferentially chosen in case of advanced peritoneal or visceral metastatic disease burden. Borderline resectable cancers with curative intent, as defined by endoscopic ultrasound, are to be routinely considered for preoperative induction therapy, in order to avoid positive margin resections. Finally, increasing use of metabolic imaging, such as through positron emission tomography, will reduce the number of patients with advanced disease and the risk for extraregional metastases undergoing curative intent operations in the future.

## Conclusion

The present study highlights some challenges in merely assigning operative treatment goals preoperatively, and achieving these goals based on postoperative review. Obviously, such assessment only considers operative and pathologic information, but not postoperative outcomes. Unfortunately, truly successful postoperative curative or palliative results are rather rare in these patients, even when an operation itself could be considered to carry this potential. Complications, recurrences, and failure to control symptoms or achieve a high level functional status are all sad reminders that an assessment based on intraoperative findings still remains rather imprecise and carries limited clinical predictive ability. Failure to achieve the intended operative goal, however, can be a useful predictor of poor outcomes, primarily the failure to cure a malignant process. In conclusion, the formulation of precise operative indications pre- and postoperatively is recommended to optimize individual outcomes and avoid unnecessary or ineffective procedures for gastrointestinal cancer. Procedures with curative intent are not supportable unless a R0 resection can be achieved. Noncurative indications should be justified based on the efficacy of symptom control, and based on the lack of less invasive alternatives.

## Competing interests

The author(s) declare that they have no competing interests.

## Authors' contributions

**RES**: conceptualized and wrote the article, did the literature search and edited the manuscript.
